# Distinct *DICER1* Hotspot Mutations Identify Bilateral Tumors as Separate Events

**DOI:** 10.1200/PO.17.00113

**Published:** 2018-04-25

**Authors:** Kenneth S. Chen, Sarai H. Stuart, Emily K. Stroup, Abhay S. Shukla, Jason Wang, Veena Rajaram, Gordan M. Vujanic, Tamra Slone, Dinesh Rakheja, James F. Amatruda

**Affiliations:** **Kenneth S. Chen**, **Sarai H. Stuart**, **Emily K. Stroup**, **Abhay S. Shukla**, **Jason Wang**, **Veena Rajaram**, **Tamra Slone**, **Dinesh Rakheja**, and **James F. Amatruda**, University of Texas Southwestern Medical Center; **Kenneth S. Chen**, **Tamra Slone**, and **James F. Amatruda**, Children’s Health Children’s Medical Center, Dallas, TX; and **Gordan Vujanic**, Sidra Medical and Research Center, Doha, Qatar.

## INTRODUCTION

*DICER1* syndrome^1^ predisposes to a variety of cancers, including pleuropulmonary blastoma (PPB),^2^ ovarian Sertoli-Leydig cell tumor (SLCT),^3^ embryonal rhabdomyosarcoma,^4^ and kidney tumors.^5-8^ In *DICER1* syndrome, most patients bear a germline null mutation in *DICER1*, and the tumors uniformly bear a second-hit missense substitution at one of five hotspot positions (1705E, 1709D, 1809G, 1810D, and 1813E).

A wide range of clinical phenotypes can be seen in *DICER1* syndrome^5,9^; some patients are asymptomatic, whereas others develop multiple tumors. In the classic cases, patients with a germline null mutation develop different somatic hotspot mutations in each tumor.^7,10-16^ However, in some patients, multiple tumors arise from germline mosaicism of the hotspot mutation, with subsequent somatic loss of the wild-type allele.^17-19^ Here we report a patient with *DICER1* syndrome who developed four tumor types at six anatomic sites over the course of 12 years. These tumors harbor four distinct hotspot mutations, which is one of the highest numbers of distinct somatic *DICER1* mutations reported in a single patient. By identifying these mutations, we show that the patient’s bilateral renal tumors and bilateral ovarian SLCTs each constituted a new primary tumor. Because we found no other mutations to explain her particularly severe clinical course, we speculate that her subsequent tumors were a product of the intense chemotherapy and radiation regimens she received.

## METHODS

Informed consent was obtained from the patient and her guardian before collection of tumor specimens. All studies were conducted after approval by a local human investigations committee and in accord with an assurance filed with and approved by the Department of Health and Human Services.

Whole-exome sequencing of tumor and germline DNA has been described previously.^20^ For archival specimens, DNA was prepared using the QIAamp DNA FFPE kit (Qiagen, Santa Clarita, CA) and amplified using the REPLI-g FFPE kit (Qiagen). Polymerase chain reaction (PCR) primers spanning exons 21 to 26 of *DICER1* are listed in Appendix Table A1. When needed, PCR products were subcloned into pCR2.1-Topo (Thermo-Fisher Scientific, Waltham, MA) before sequencing.

For *TP53* sequencing, coding regions were amplified using the Accel-Amplicon Comprehensive *TP53* Panel (Swift Biosciences, Ann Arbor, MI). Sequencing with the Illumina MiSeq Nano v2 kit (Illumina, San Diego, CA) generated 1.5 million paired-end reads. Reads were trimmed using trimmomatic^21^ in the Galaxy Project and were aligned to human genome GRCh38 using BWA-MEM^22^; 88% mapped to the *TP53* locus, producing a mean depth of 13,367x in targeted regions. Variants were called using the FreeBayes algorithm v0.9.20 using frequency-based pooled calling instead of simple diploid calling, which allowed the algorithm to detect subclonal mutations.^23^

## RESULTS

### Clinical Presentation

**Fig 1.**
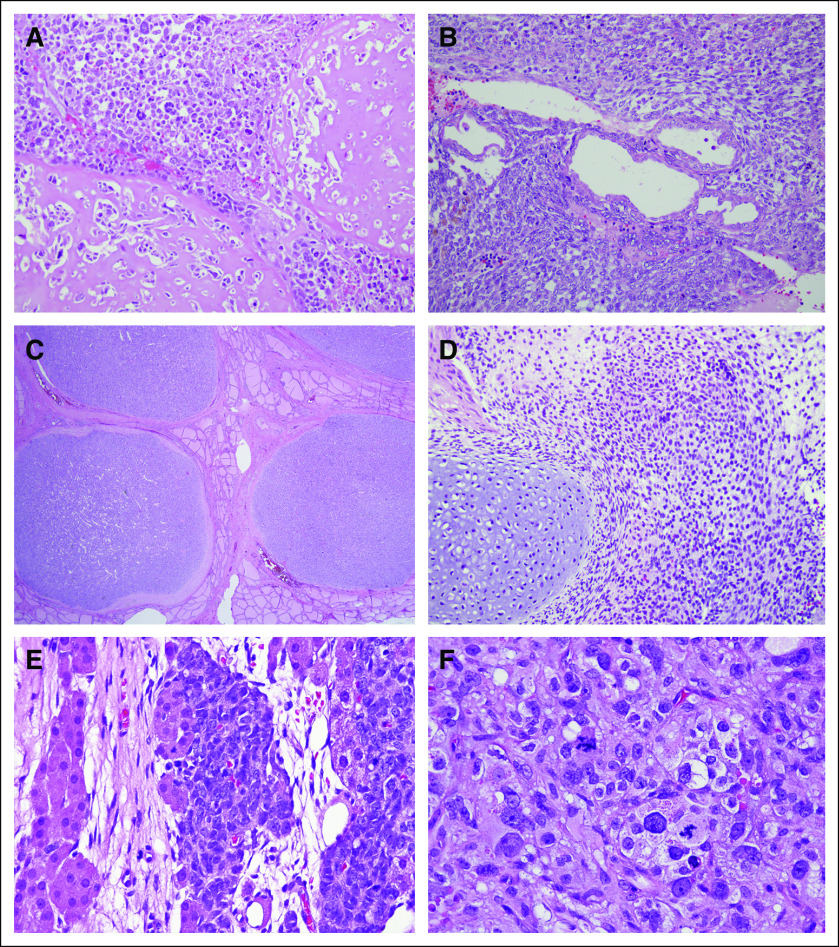
Histology of tumors. (A) Left kidney tumor (magnification, ×20). (B) Right kidney tumor (magnification, ×20). (C) Thyroid nodules (magnification, ×2). (D) Rhabdomyosarcoma arising from bladder (magnification, ×20). (E) Sertoli-Leydig cell tumor arising from right ovary (magnification, ×40). (F) Anaplastic elements of Sertoli-Leydig cell tumor arising from right ovary (magnification, ×40).

The patient (CMCW11) presented originally at 5 years of age with a left kidney mass. She had no family history of cancer. She underwent left radical nephrectomy with lymph node resection. At the time, she was diagnosed with diffusely anaplastic Wilms tumor with extensive rhabdomyoblastic, osteoblastic, and chondroblastic differentiation (Fig 1A). This diagnosis was confirmed by central pathologic review. She had lymph node, pulmonary, and rib metastases. She was treated with 10 months of combination chemotherapy (vincristine, doxorubicin, cyclophosphamide, irinotecan, and etoposide) targeted at both anaplastic Wilms tumor and sarcoma, together with 2,100 cGy whole-abdomen irradiation given in 14 fractions over 19 days.

When the patient was 10 years old, a new mass arose in her remaining kidney, with a histology similar to her prior tumor, and she was diagnosed with relapsed Wilms tumor (Fig 1B). She initially received ifosfamide, carboplatin, and etoposide, but because of toxicity she was subsequently switched to vincristine, doxorubicin, cyclophosphamide, ifosfamide, and etoposide. On completion, she underwent right radical nephrectomy and started hemodialysis.

This patient’s kidney tumors were diagnosed initially as Wilms tumor, but in retrospect their unique histologic features are more consistent with anaplastic sarcoma of the kidney. This entity was first described in 2007, after our patient’s kidney tumor first arose.^24^ Biallelic *DICER1* mutations have been seen in both Wilms tumor and anaplastic sarcoma of the kidney, which may represent neoplastic degeneration from cystic nephroma.^6-8^

At 12 years of age, the patient developed multiple nodules throughout her thyroid. Thyroidectomy revealed follicular adenomas (Fig 1C).

At 13 years of age, a mass arising from her bladder was diagnosed as embryonal rhabdomyosarcoma with focal cartilaginous differentiation (Fig 1D). She was treated with vincristine, dactinomycin, and cyclophosphamide and a radical cystectomy.

At 15 years of age, she developed ovarian masses and underwent sequential salpingo-oophorectomies. Both ovaries harbored SLCTs with intermediate to poor differentiation (Figs 1E and 1F). One of the tumors had anaplastic sarcomatoid foci. Since that time, she has been observed for > 2 years, with no further disease.

### DICER1 Mutations

**Fig 2.**
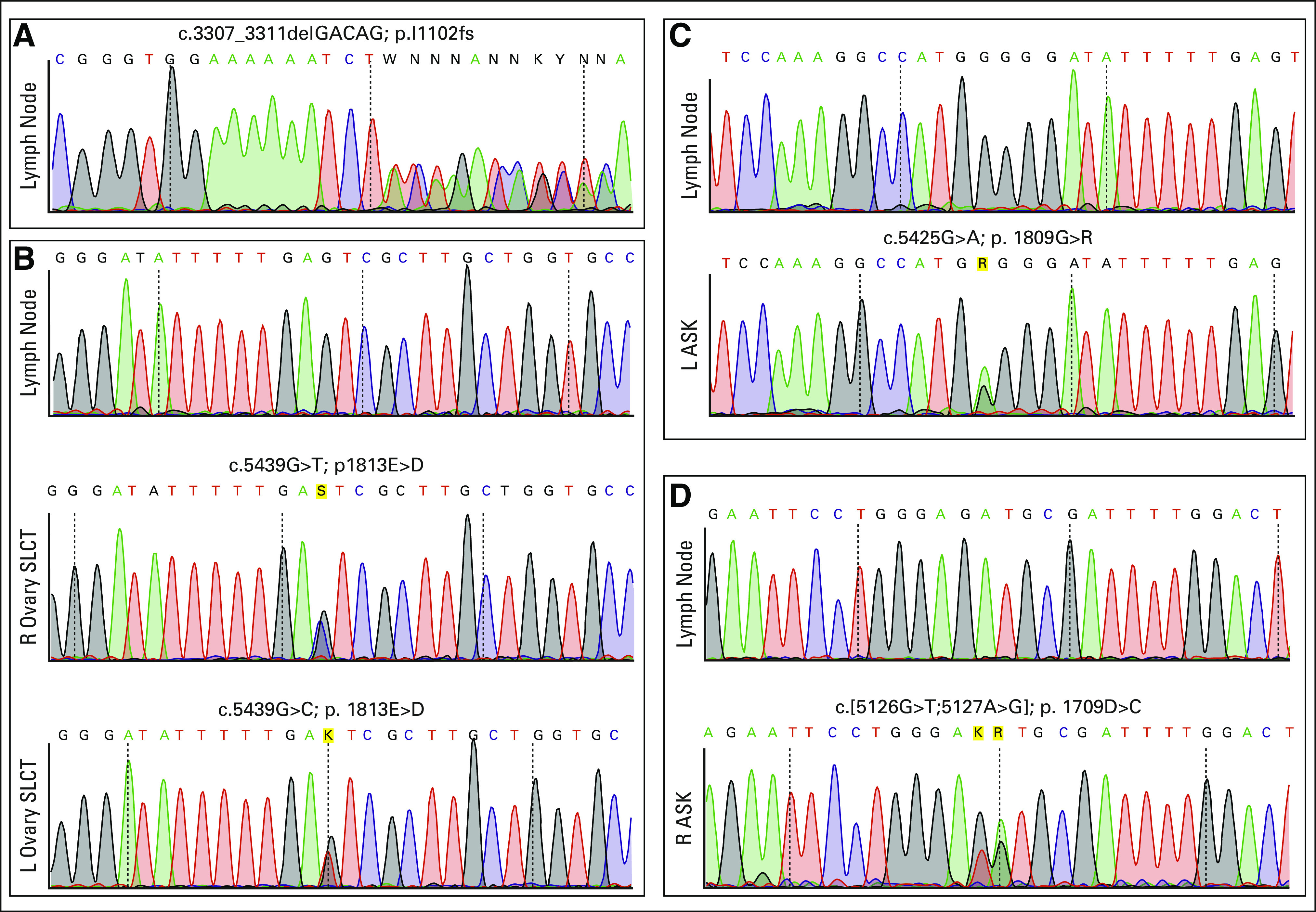
Sanger sequencing chromatograms demonstrating the patient’s (A) *DICER1* germline frameshift mutation and (B) RNase IIIb hotspot mutations in her ovarian Sertoli-Leydig cell tumors (SLCTs); (C) left (L) kidney tumor; and (D) right (R) kidney tumor. ASK, anaplastic sarcoma of the kidney.

We performed whole-exome sequencing on the patient’s first kidney tumor and germline DNA as part of a retrospective Wilms tumor sequencing project.^20^ Her germline harbored a frameshift deletion (c.3307_3311delGACAG, p.Ile1102fs; Fig 2). Her tumor bore an additional somatic hotspot mutation (c.5425G>A; p.G1809R; Fig 2 and Table 1).

**Table 1.**
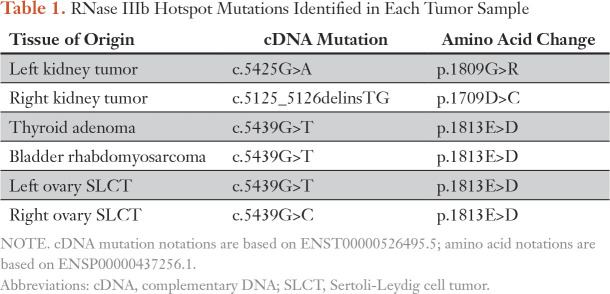
RNase IIIb Hotspot Mutations Identified in Each Tumor Sample

**Fig A1.**
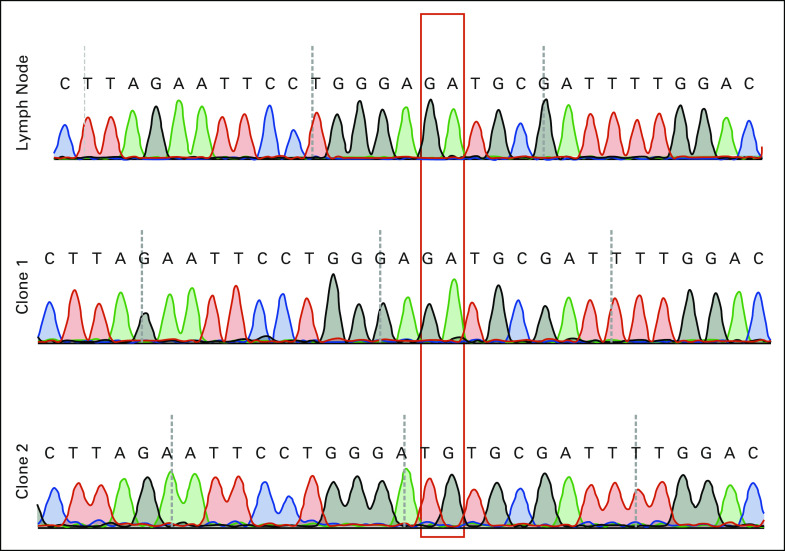
Sequence of the D1709 region in normal lymph node and in representative clones of the amplicon from the right kidney tumor, demonstrating that the c.G5126T and c.A5127G mutations are on the same DNA strand.

We then sequenced the RNase III domains of *DICER1* in archival specimens from her other tumors. Her contralateral renal tumor exhibited a different hotspot mutation, c.5125_5126delinsTG; p.D1709C (Fig 2 and Table 1). Because this change spanned two nucleotides, it was possible that the two nucleotide variants (c.G5125T and c.A5126G) occurred on separate strands. To sequence strands separately, we subcloned the PCR amplicon spanning this mutation and found that both single-nucleotide variants occurred on the same strand, making the tumor heterozygous for a single somatic hotspot mutation (Appendix Fig A1). Contrary to our clinical suspicion at the time, her contralateral kidney tumor was, in fact, a second primary.

We also sequenced *DICER1* from her thyroid follicular adenoma and bladder rhabdomyosarcoma. Both showed c.G5439T (p.E1813D) mutations (Table 1). *DICER1*-associated ovarian and cervical embryonal rhabdomyosarcomas frequently exhibit cartilaginous differentiation,^11,25^ and her bladder tumor may be a related tumor. Bladder rhabdomyosarcomas have been associated previously with *DICER1* syndrome, although specific discussion of their histologic features was not reported.^4^

Her two ovarian tumors were detected at the same time, and it was unclear whether they represented distinct primary tumors or metastatic spread. The ovarian tumors harbored different mutations at the same position: c.G5439T and c.G5439C (Fig 2 and Table 1), indicating that they arose independently.

### TP53 Status

In many tumors, *DICER1* mutations are associated with *TP53* inactivation,^20,26,27^ but clinical sequencing of our patient’s ovarian tumor did not detect any mutations in *TP53*. Because her phenotype was so severe, we performed targeted *TP53* sequencing in her other tumor samples (Appendix Table A2). Her initial renal tumor bore a p.V274L variant, as we described previously.^20^ However, her remaining tumors had no additional somatic *TP53* variants and did not undergo loss of heterozygosity at *TP53*.

## DISCUSSION

Clinically, the discovery of a second tumor with similar histology can raise the question of whether it is a recurrence or a new primary tumor. The answer has important therapeutic implications; new primary tumors are treated with front-line therapy to which recurrent tumors rarely respond. In this patient, the bilateral renal and SLCTs were suspected to represent metastatic spread. The differing *DICER1* mutations were identified retrospectively. Had we known that her second kidney tumor was, in fact, metachronous, she might have avoided the increased toxicity of chemotherapy regimens designed for relapsed or refractory tumors. Similarly, when she was diagnosed with bilateral ovarian tumors, we were concerned that she might need aggressive therapy for eradicating hematogenous metastases. In fact, the cartilaginous elements of her bladder tumor led us to question whether it could instead represent a distant recurrence of her renal sarcoma, although its distinct *DICER1* mutation proved its independence.

Thus, this case highlights a powerful but often overlooked application of clinical tumor sequencing: better understanding of the biology behind tumor formation. We used sequencing here to show that the contralateral tumors arose independently. As sequencing costs fall, clinicians should be cognizant that tumor sequencing can help distinguish between new primary tumors and recurrences.

In this case, sequencing at initial diagnosis would have also had the added benefit of alerting clinicians to her potential *DICER1* status. In the case of *DICER1* syndrome, because second-hit mutations occur at such stereotyped positions, sequencing separate tumors could even occur by simple Sanger sequencing rather than by next-generation sequencing. In patients with *DICER1* syndrome, dedicated tests may be developed for identifying these stereotyped mutations, perhaps including circulating tumor DNA assays as a noninvasive screening method.

This is one of the most severe cases in the literature of multiple distinct somatic *DICER1* mutations arising in a patient. Germline whole-exome sequencing did not detect any other known cancer-predisposing mutations. Although we cannot rule out other secondary mutations, targeted *TP53* sequencing did not reveal *TP53* inactivation in her subsequent tumors. We speculate that the particular severity of her clinical course could have been related to the intense chemo- and radiotherapy she received at her initial cancer diagnoses. A few case reports have described patients with PPB/*DICER1* syndrome who developed one or more cancers after alkylating chemotherapy (Table 2). However, these cases constitute a small sample size and are likely the most extreme cases, highlighted because of publication bias. Ongoing prospective studies such as the International PPB Registry are necessary to study the long-term effects of chemotherapy and radiation in patients with *DICER1*.

**Table 2.**
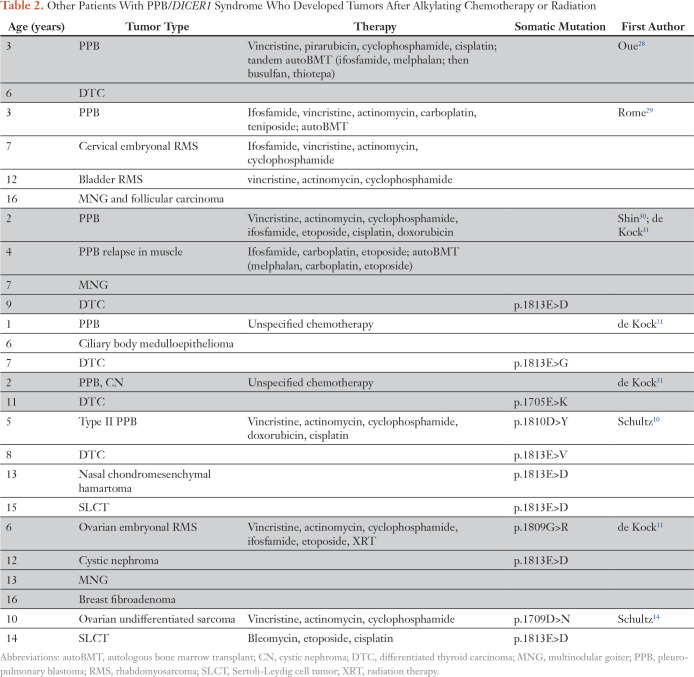
Other Patients With PPB/*DICER1* Syndrome Who Developed Tumors After Alkylating Chemotherapy or Radiation

## Figures and Tables

**Table A1.**
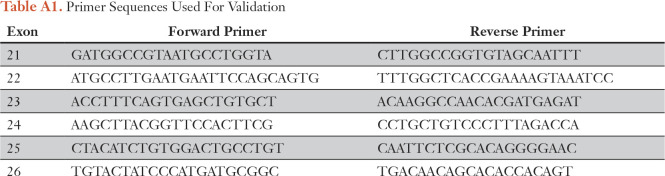
Primer Sequences Used For Validation

**Table A2.**
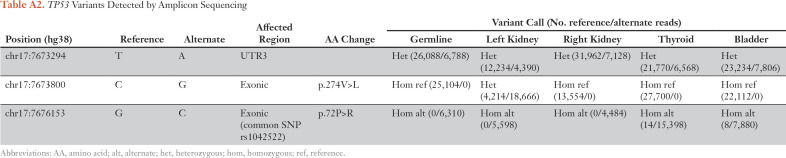
*TP53* Variants Detected by Amplicon Sequencing
